# The effects of HIV and systolic blood pressure on mortality risk in rural South Africa, 2010–2019: a data note

**DOI:** 10.1186/s13104-023-06478-w

**Published:** 2023-09-12

**Authors:** Brian Houle, Samuel J Clark, Chodziwadziwa W Kabudula, F Xavier Gómez-Olivé, Nicole Angotti, Enid Schatz, Andrea M Tilstra, Sanyu A Mojola, Jane Menken

**Affiliations:** 1 School of Demography, The Australian National University, Canberra, Australia; 2 MRC/Wits Rural Public Health and Health Transitions Research Unit (Agincourt), Faculty of Health Sciences, School of Public Health, University of the Witwatersrand, Johannesburg, South Africa; 3 Department of Sociology, The Ohio State University, Columbus, OH USA; 4 Department of Sociology, American University, Washington, D.C USA; 5 Department of Public Health, University of Missouri, Columbia, MO USA; 6 Leverhulme Centre for Demographic Science, Nuffield Department of Population Health, and Nuffield College, University of Oxford, Oxford, UK; 7 Department of Sociology, School of Public and International Affairs, and Office of Population Research, Princeton University, Princeton, NJ USA; 8 Institute of Behavioral Science, University of Colorado Boulder, Boulder, CO USA

**Keywords:** Mortality, Rural, South Africa, HIV, Hypertension, Blood pressure, Morbidity, Ageing

## Abstract

**Objectives:**

South Africa is experiencing both HIV and hypertension epidemics. Data were compiled for a study to identify effects of HIV and high systolic blood pressure on mortality risk among people aged 40-plus in a rural South African area experiencing high prevalence of both conditions. We aim to release the replication data set for this study.

**Data description:**

The research data comes from the 2010-11 Ha Nakekela (We Care) population-based survey nested in the Agincourt Health and socio-Demographic Surveillance System (AHDSS) located in the northeast region of South Africa. An age-sex-stratified probability sample was drawn from the AHDSS. The public data set includes information on individual socioeconomic characteristics and measures of HIV status and blood pressure for participants aged 40-plus by 2019. The AHDSS, through its annual surveillance, provided mortality data for nine years subsequent to the survey. These data were converted to person-year observations and linked to the individual-level survey data using participants’ AHDSS census identifier. The data can be used to replicate Houle et al. (2022) — which used discrete-time event history models stratified by sex to assess differential mortality risks according to Ha Nakekela measures of HIV-infection, HIV-1 RNA viral load, and systolic blood pressure.

## Objective

South Africa is experiencing twin HIV and hypertension epidemics. As antiretroviral therapy has become widespread, resulting survival gains and ageing of people living with HIV have highlighted the importance of understanding emerging morbidity and mortality patterns among people living with HIV [1–3]. The South African burden of noncommunicable diseases (NCD) – particularly hypertension – is also high [4, 5]. A 2022 study examined associations between HIV and mortality, blood pressure and mortality, and joint associations between HIV and blood pressure and mortality over an eight-year period [6]. The data set we have released permits replication of the study findings and further investigation of effects of these epidemics, individually and in interaction, on mortality at ages 40-plus.

This data set was constructed as part of a larger project – HIV after 40 in rural South Africa: Aging in the context of an HIV epidemic (HIV40) – that more broadly examines life course and contextual variation in HIV risk and protective behaviors among middle-aged and older adults in a rural sub-Saharan African population [6–20]. The website https://hivafter40.princeton.edu contains information on all aspects of the overall study including survey instruments (see Table 1:Data file 1), links to publications, etc. The study includes data from the AHDSS, the nested quantitative Ha Nakekela (“We Care”) Survey and the qualitative longitudinal Izindaba za Badala Study (‘matters that concern older people’), nested within the survey. Please see https://hivafter40.princeton.edu/data for more information.

## Data description

Analyses in the paper *Twin epidemics: The effects of HIV and systolic blood pressure on mortality risk in rural South Africa, 2010–2019* [6] can be replicated using the data set “HIVafter40_Twin_Epidemics_HIV_NCD.dta” deposited in the DataFirst repository, 10.25828/vr0p-ch08. The Table 1: Data file 2 file describes all available files.

**Table 1:** Overview of data files/data sets

Label	Name of data file/data set	File types (file extension)	Data repository and identifier (DOI or accession number)
Data file 1	Tesa-2010-2019-instruments	Zip (.zip)	DataFirst 10.25828/vr0p-ch08 [27]
Data file 2	tesa-2010-2019-project-description	PDF (.pdf)	DataFirst 10.25828/vr0p-ch08 [27]
Data file 3	ahdss-hanakekela-hiv-hyp-codebook	PDF (.pdf)	DataFirst 10.25828/vr0p-ch08 [27]
Data file 4	Tesa-2010-2019-dofiles	Zip (.zip)	DataFirst 10.25828/vr0p-ch08 [27]
Data file 5	hivafter40-twin-epidemics-hiv-hyp-codebook	PDF (.pdf)	DataFirst 10.25828/vr0p-ch08 [27]
Data set 1	Tesa-2010-2019-v1	Zip (.zip)	DataFirst 10.25828/vr0p-ch08 [27]

The data set was compiled from two sources.

- The **AHDSS** is conducted by the MRC/WITS Agincourt Research Unit which is affiliated with both the University of the Witwatersrand and the South African Medical Research Council [21]. Its website provides information on research, publications, and data access for the extensive range of studies carried out in the research site: https://www.agincourt.co.za. Censuses that have been carried out annually since 1992 provide vital event and sociodemographic information on households and residents. Records for an individual are linked to provide a longitudinal history for each person. The censuses also serve as sampling frames for more detailed studies carried out in the Agincourt study site. For all respondents included in the HIV and NCD Survey (described next), sociodemographic information was extracted from the AHDSS.
- The **Ha Nakekela HIV and NCD Survey**, carried out in 2010-11, is based on an age-sex stratified random sample of people aged 15-85-plus selected from the 2009 AHDSS Census. To date, a number of studies have utilized this survey [6, 8–12, 16, 17, 20, 22–26]. HIV and NCD biometric data (see [22, 24] for descriptions of the biometric data collection) and a behavioral survey on sexual behavior and other health practices were collected. Of the 7,662 people originally selected, 4,362, including 3,024 aged 40-plus, consented to be interviewed, tested for HIV and have blood pressure measured. Gómez-Olivé et al. [24] reported extremely high HIV prevalence in this population, and Clark et al. [22] showed a high burden of hypertension.

The **HIV40 Study** linked information from Ha Nakekela to AHDSS mortality records through mid-2019 (see Data file 3) using respondents’ AHDSS census identifier. For each of the 7,662 people in the Ha Nakekela original sample, we calculated the survey weight as the inverse of the probability that the person was selected for the sample. To adjust for survey nonresponse, we used a logistic regression model of factors from the 2009 census to predict the probability of participation and its inverse, the inverse probability sampling weight (IPSW). Final weights for participants are the product of the IPSW and the survey weight [6, 20]. For the replication data set (Data set 1), some variables were dropped and some variables recoded (see Data file 4 and Data file 5). All observations were converted to person-year observations – yielding one person-year for each age in which respondents were followed in the AHDSS census. Only person-years when the participant was aged 40-plus were retained. We then calculated discrete-time models of the risk of dying based on HIV and blood pressure characteristics, adjusted for 2009 Census covariates (see Data file 4; [6]).

### Limitations

HIV status and systolic blood pressure were only measured at baseline. Subsequent follow-up surveys or linkage with other data such as clinic records would permit a more detailed understanding of how risk factors and changes in those risk factors are associated with mortality change over time. While mortality was high in this population, given the limited sample size there were too few deaths (n = 477) to include cause of death information.

## Data Availability

The data described in this Data note can be accessed on the DataFirst repository under (10.25828/vr0p-ch08) [27]. Please see Table 1 for details and links to the data. Access to Data set 1 requires registration with DataFirst and an application for access to a licensed data set. https://www.agincourt.co.za contains information on access to additional studies conducted in the AHDSS and a complete list of publications. https://hivafter40.princeton.edu contains copies of all study protocols and data instruments and a complete list of publications related to the HIVafter40 Study.

## Abbreviations

AHDSS: Agincourt Health and Demographic Surveillance System
NCD: Noncommunicable disease
